# Twin-peak temporal regulation during human neocortical development

**DOI:** 10.1038/s41421-019-0129-3

**Published:** 2019-12-17

**Authors:** Wei Wang, Guang-Zhong Wang

**Affiliations:** CAS Key Laboratory of Computational Biology, CAS-MPG Partner Institute for Computational Biology, Shanghai Institute of Nutrition and Health, Shanghai Institutes for Biological Sciences, University of Chinese Academy of Sciences, Chinese Academy of Sciences, Shanghai, 200031 China

**Keywords:** Transcriptomics, Transcription

## Abstract

Understanding the temporal and spatial expression patterns of the human cerebral cortex is essential for expanding knowledge of its functionality. Previous analysis focused on the differentially expressed genes (DEGs) among cortical subregions revealed an hourglass model for interareal differences. However, the overall pattern of transcriptional differences during the development of every region remains to be fully explored. Here, analysing more than 800 neocortex samples from lifespan transcriptional profiles revealed that excitatory neurons are more regulated than inhibitory neurons in the foetal brain. Developmental DEGs tend to be resting state or memory encoding-related and are also involved in autism and Alzheimer’s disease. In addition, twin peaks of DEGs occur during the development of each neocortex region, with a first peak appearing in the perinatal period and an unexpected second peak appearing around childhood. Genes in these peaks have similar functions, but the second peak is more inhibitory neuron related. All these results emphasize the significance of this unique temporal regulatory pattern for human neocortical development.

## Introduction

The human cerebral neocortex is formed through development and involved in various complex behaviours. However, the complete development pattern of each cortical region has not been fully explored. Understanding the development patterns of human brain regions at the molecular level is of vital importance as it may provide a more comprehensive view of the uniqueness of the human brain^1–5^. Due to advancements in microarray and next-generation sequencing technology, most transcriptional activities of mRNAs can be accessed in different brain regions at relatively low cost. These technologies revealed that 86% of genes are expressed in the primate brain, and ~82% of genes are expressed in the cerebral neocortex^6–10^.

Examining the transcriptional activities in multiple brain regions across the lifespan has demonstrated that the regulation of the brain regional transcriptome is not evenly distributed among different stages^6,7,10^. This non-uniformity exists both spatially and temporally. Specifically, a large number of genes are upregulated and downregulated during prenatal development^10^. In the human prefrontal cortex, the regulation of gene expression is quicker during foetal development than during any other developmental stage, and the speed is decreased in infancy or later period of life^7^. Surprisingly, by comparing the number of differentially expressed genes (DEGs) among 11 cortex regions, an hourglass model was proposed, which indicates that the transcriptional divergence among different subcortical regions is more significant in early and late periods of development than in childhood^11^. This model provides the basis of our understanding of developmental differences in different human neocortical regions^12^. However, whether a unified model exists for the global regulation pattern of a particular neocortex region during its development remains unclear, let alone whether different neocortex areas follow the same pattern across the lifespan.

Here, by investigating the human neocortex transcriptome data across the whole lifespan, we assessed the number of DEGs during development in each of the 11 regions. The neocortex regions exhibited a waterfall mode regarding the overall developmental dissimilarity. Additionally, the number of developmental DEGs peaked in both the perinatal period and around the childhood period. Then, we systematically characterized the features of these peaks, which revealed the crucial regulatory periods of different neocortex regions.

## Results

### Transcriptional differences in the human neocortex among different developmental stages exhibit a waterfall mode

To characterize the global transcriptional regulation pattern per neocortex subregion, we collected 886 samples of brain regional expression data from the Human Brain Transcriptome (HBT) database^10^, which spans from 10 weeks post conception (pcw) to 82 years of age. In total, 11 brain regions were used for downstream analysis. These regions included the frontal lobe: the orbital (OFC), dorsolateral (DFC), ventrolateral (VFC), medial (MFC), and primary motor (M1C) cortices; the parietal lobe: the primary somatosensory (S1C) and posterior inferior (IPC) cortices; the temporal lobe: the primary auditory (A1C), posterior superior (STC), and anterior inferior (ITC); and the occipital lobe: the primary visual (V1C) cortex, as previously described^11^. Similar to previous methodology^11^, ANOVA was performed to identify temporal DEGs during development for each neocortex region. Our results show that 11,771 genes were differentially expressed between any two developmental stages (stages were defined as described in ref. ^10^, and the samples were divided by the same stage criteria as previously reported) in at least one neocortical region (Supplementary Table S1), which accounted for 85% of all 13,834 expressed genes, suggesting that these neocortex regions are heavily regulated during brain development.

**Fig. 1:**
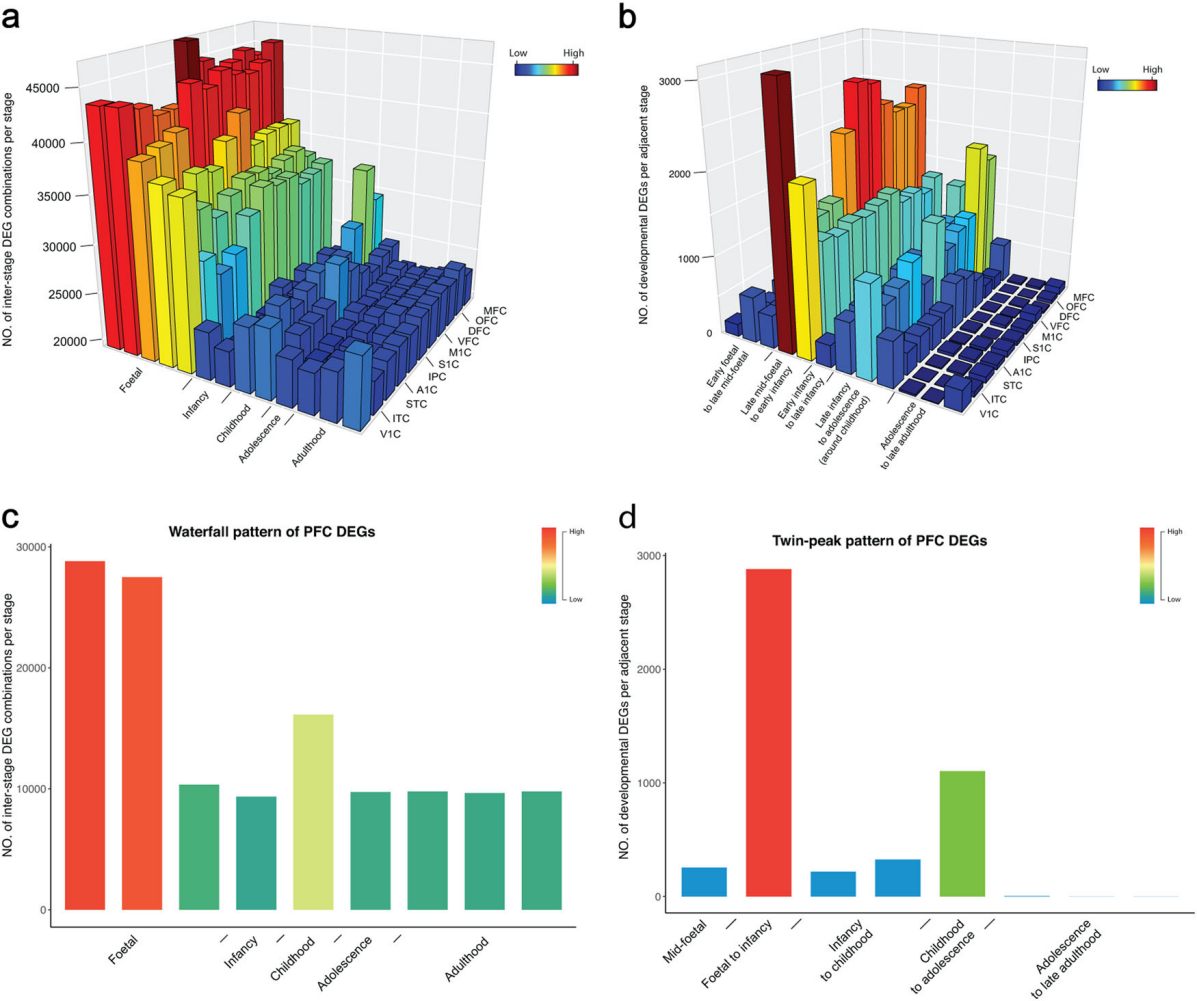
Waterfall mode and twin-peak regulation of neocortical development. **a** 3D heatmap shows the number of temporal DEG combinations of 11 neocortex subregions. **b** The number of adjacent developmental DEGs per neocortex subregion. **c** Temporal DEG combinations of the human prefrontal cortex, which is from an independent dataset. **d** The number of adjacent developmental DEGs of the human prefrontal cortex. Different colours represent the number of DEGs detected from high (red) to low (blue)

Tukey’s pairwise comparison estimated the contribution of DEGs in a specific developmental stage^11^. The inter-stage transcriptional divergence in each brain region exhibited a waterfall pattern. For all neocortex subregions, early and mid-foetal periods exhibited the most prominent dissimilarity, with a sharp decrease at the neonatal stage (Fig. 1a). This waterfall pattern of temporal divergences in late foetal and early infancy suggests that the perinatal period is an essential developmental stage that represents dramatic changes in gene expression globally in human neocortex regions.

### Twin-peak pattern of gene regulation across developmental stages

DEGs in adjacent developmental stages (developmental DEGs) were calculated (from stage 3 to 15) to further investigate how the brain transcriptome changes stage by stage. Unexpectedly, the number of developmental DEGs burst twice across the whole lifespan in the neocortex (Fig. 1b and Supplementary Table S2). The first peak appeared in the perinatal period (from late mid-foetal to early infancy), while the striking second peak occurred around the period of childhood (from late infancy to adolescence). The first and second peaks involved more than 6,000 and 4,500 DEGs in each neocortex subregion, respectively, which implies that two critical regulatory periods exist at the molecular level during human neocortex development and maturation, with the most complex regulation occurring in the perinatal brain^6,7,10^. In addition, the pattern was robust when we performed a permutation test by randomly choosing the same number of samples from each stage (Supplementary Fig. S1a, b). By contrast, we found no similar pattern when we estimated the distribution of developmental DEGs in other non-neocortical regions (including the hippocampus, amygdala, striatum, cerebellar cortex and mediodorsal nucleus of the thalamus) from the same database (Supplementary Fig. S1c, d), suggesting that the twin-peak mode is a unique regulation pattern of neocortex development.

### Replication of twin-peak regulation in human prefrontal cortex transcriptome

Exploring regulation patterns during development might be difficult as developmental data points are often incomplete, especially in the early developmental stages. Therefore, we sought to validate the twin-peak pattern observed in human neocortex regions by utilizing an independent dataset, i.e., human prefrontal cortex transcriptome data, which contain 269 samples across the lifespan^7^. As expected, although the sampling time points of the two datasets were slightly different, both the waterfall model and twin-peak pattern were well replicated in the independent dataset (Fig. 1c, d). In this prefrontal cortex dataset, 2,880 DEGs were detected in the first peak, which is twice the number of DEGs detected in the second peak (1103 DEGs; Supplementary Table S3). All these results illustrate the robustness of the twin-peak pattern observed.

### Shared developmental DEGs among neocortex subregions

**Fig. 2:**
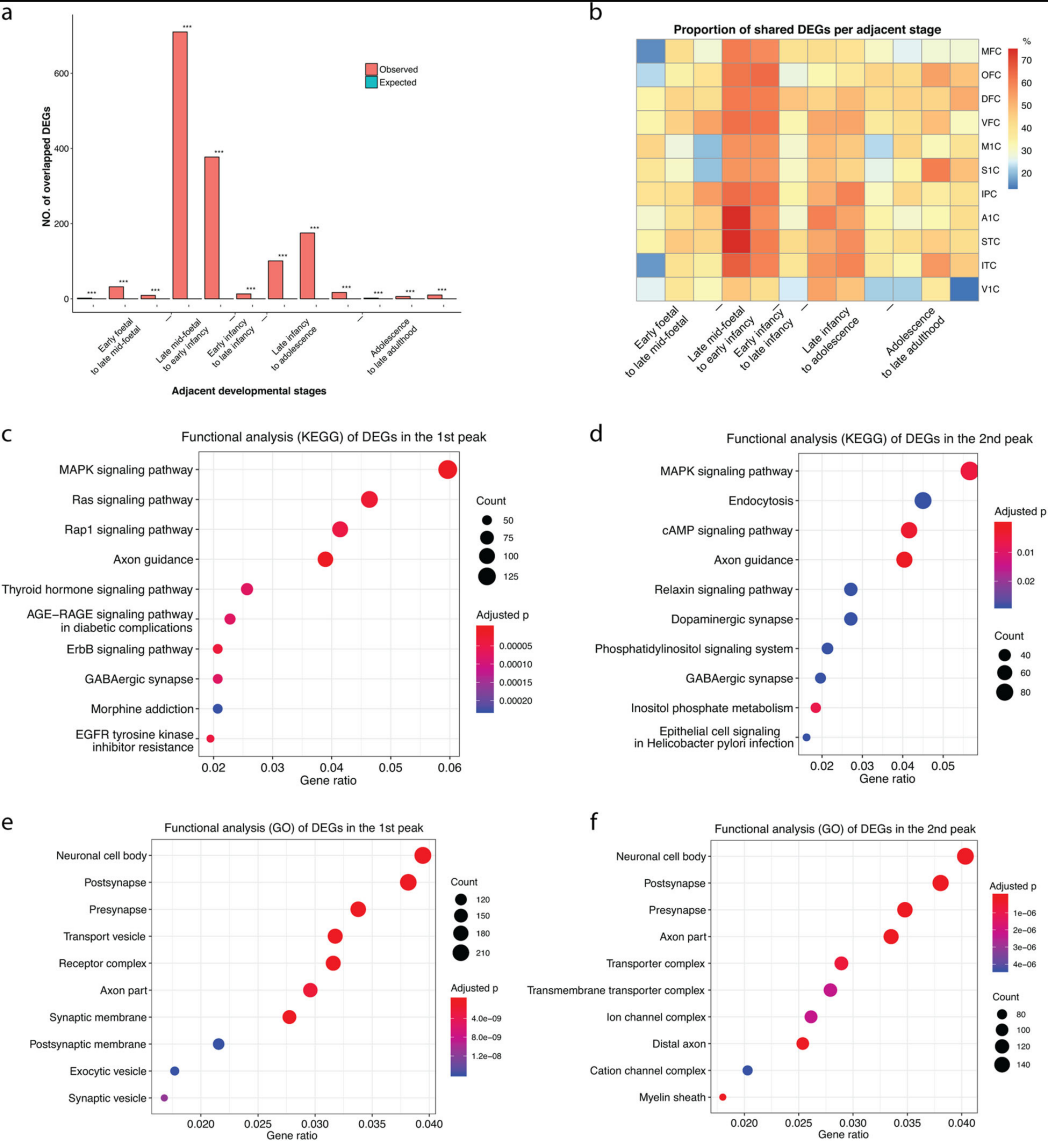
Characteristics of DEGs in twin-peak regulation. **a** Number of overlapping DEGs across 11 neocortex subregions. Red bars represent the observed number of overlapped DEGs. Green bars represent the number of overlapped DEGs detected in the permutation experiments (10,000 times). Asterisks on the bar plot represent the significant level from the permutation test. *P < 0.05, **P < 0.01, ***P < 0.001. **b** Heatmap of the proportion of developmental DEGs in a neocortex subregion shared with other subregions. **c**, **d** Enrichment of KEGG pathways of the first peak (**c**) and second peak (**d**) DEGs. **e**, **f** Enrichment of gene ontology terms (GO) of the first peak (**e**) and second peak (**f**) DEGs. The size of the circle represents the number of DEGs in each category, and the colour of the circle represents the significant level of enrichment

To examine the distribution of developmental DEGs, we surveyed whether the same DEGs tended to be regulated in multiple brain areas at the same stage. DEGs in the twin peaks significantly overlapped across neocortex regions in 10,000 permutation experiments (Fig. 2a, *P* < 0.001 for all 12 comparisons), and the proportion of DEGs shared by different neocortex subregions in the peak periods was higher than that in other developmental periods (Fig. 2b). Additionally, 70% of DEGs (2,886 genes) in the second peak were differentially regulated in the first peak period (Supplementary Table S4), suggesting significant similarities in the regulatory networks during the two critical periods.

Then, we investigated the functionality of the DEGs in those peak periods. KEGG pathway analysis showed that the DEG lists of the twin-peak periods were both significantly enriched in axon guidance, GABAergic synapse and MAPK signalling pathway (first peak: Benjamini–Hochberg corrected *P* < 7.39 × 10^−5^; second peak: Benjamini–Hochberg corrected *P* < = 0.029; Fig. 2c, d). In addition, DEGs in the first peak were involved in morphine addiction (Benjamini–Hochberg corrected *P* = 2.3 × 10^−4^) and pathways of cell proliferation, growth or migration, such as the Ras signalling pathway and ErbB signalling pathway (Benjamini–Hochberg corrected *P* < 3.67 × 10^−5^), which is related to the increase in brain size. DEGs in the second peak were mainly involved in Dopaminergic synapse (Benjamini–Hochberg corrected *P* = 0.029), a pathway related to learning and memory^13,14^, which suggests that the regulation of this peak might play a role in the formation of life experience. Similarly, GO analysis further showed that both peaks were enriched in the neuronal cell body, axon part, presynapse and postsynapse (Benjamini–Hochberg corrected *P* < 8.24 × 10^−8^; Fig. 2e, f). More interestingly, DEGs only in the first peak tended to be involved in axon and dendrite development (Benjamini–Hochberg corrected *P* < 9.56 × 10^−04^; Supplementary Fig. S2), suggesting that extensive neuronal developmental-related regulation occurs during the prenatal period of these neocortex regions. By contrast, no significant enrichment of pathways was found for DEGs only in the second peak.

### More developmental DEGs are detected than interregional DEGs

We further compared the distribution of developmental DEGs with previously identified brain regional DEGs^11^. First, there were, on average, four-fold more developmental DEGs than interregional DEGs (11,610 developmental DEGs vs. 3,133 interregional DEGs), indicating that the developmental regulation of each neocortex period involves a large number of molecules and pathways and is more complicated. Second, the number of developmental DEGs detected in the early foetal brain was less than one-third that of regional DEGs (531 developmental DEGs vs. 1,785 regional DEGs). Third, a group of developmental DEGs in the twin-peak regulation periods overlapped with brain regional DEGs in the same periods (Supplementary Table S5), indicating that those genes are participating in both neocortex developmental and regional differentiation, which may promote the functional synchronization of different neocortex regions^11^.

### Enrichment of brain resting state and memory encoding-related genes in developmental DEGs

**Fig. 3:**
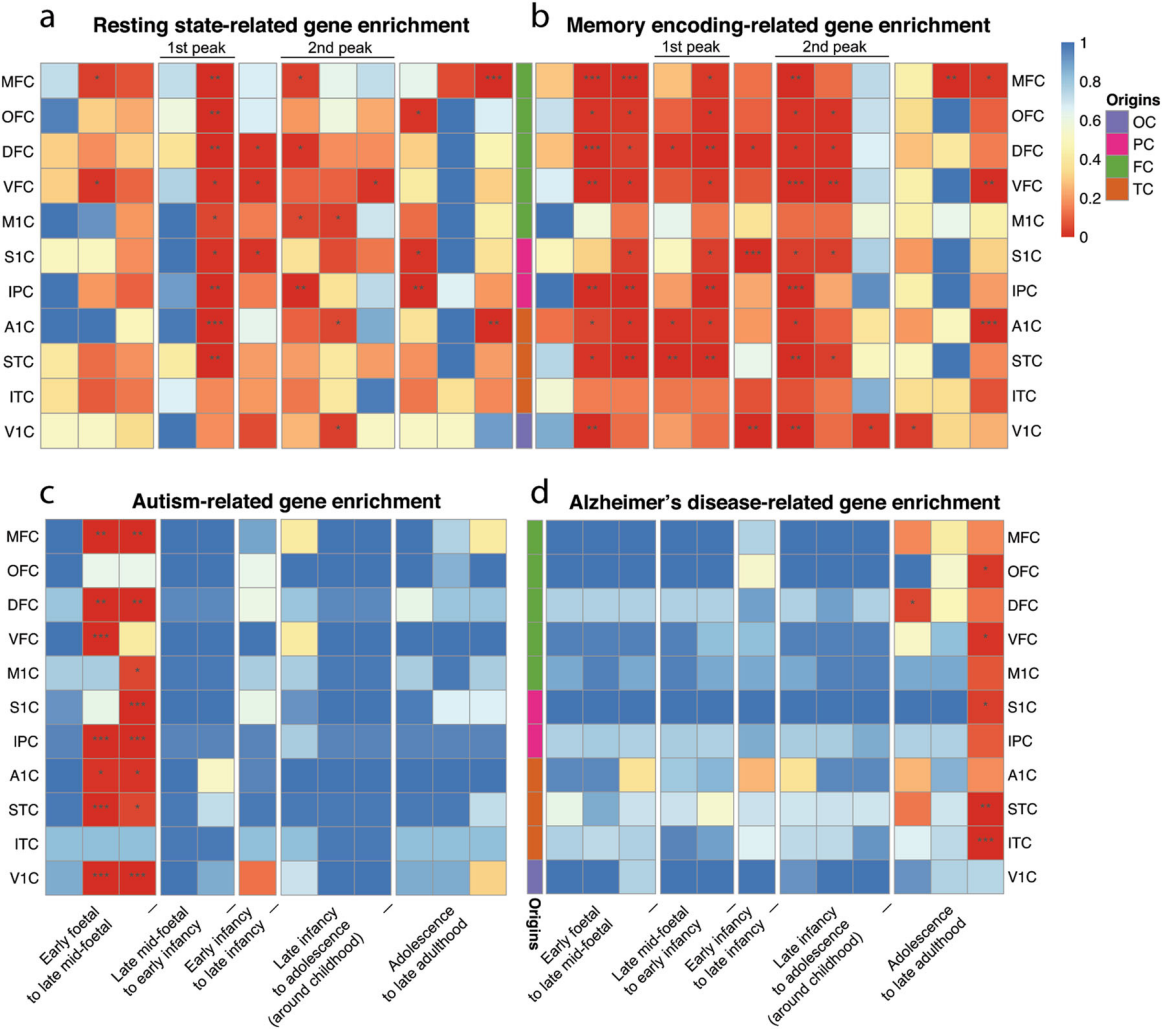
Enrichment of various brain functional gene sets with developmental DEGs. **a** Resting state-related genes are enriched at the first peak period and thereafter in most neocortex subregions. **b** Human memory encoding-related genes are enriched in multiple periods of the lifespan. **c** Autism-related genes are mainly enriched in different neocortex regions of mid-foetal stages. **d** Alzheimer-related genes are mainly enriched in late adulthood. Twin-peak periods and the origin of these neocortical regions are indicated in the plot. Asterisks on each cell of the heatmap represent the significance level (*P* from Fisher’s exact test, Benjamini–Hochberg corrected). *P < 0.05, **P < 0.01, ***P < 0.001

Based on recent evidence, the brain’s resting state networks can be detected in the early development of the brain^15,16^, indicating the possibilities that their molecular activities and functional networks coordinate developmentally. A recently identified group of genes whose expression profiles are related to the brain’s resting state signal has provided valuable clues for further investigation of the genetic basis of advanced brain functions^17,18^. Thus, we explored the regulation patterns of these genes during development. Eighty-seven of the 168 (>50%) collected resting state-related genes are differentially expressed in at least one neocortex during development. Additionally, resting state-related genes are enriched in DEGs in 9 of the 11 neocortex regions during the first peak period (Benjamini–Hochberg corrected *P* < 0.05; Fig. 3a). Similarly, we found significant enrichment of our recently identified memory encoding-related genes^19^ with those developmental DEGs. Furthermore, those memory encoding-related genes tend to be differentially regulated primarily at the twin-peak periods, as well as at mid-foetal and late adulthood periods, in most neocortex regions (Benjamini–Hochberg corrected *P* < 0.05; Fig. 3b). Generally, compared with the brain resting state-related genes, our previously identified memory encoding genes tend to be differentially regulated at more developmental stages, especially in foetal brain development, indicating the importance of regulating memory-related genes during neocortical development. Indeed, mapping the genes with GO annotations of “learning or memory” and “cognition” to the DEGs across different developmental stages revealed a similar widespread distribution pattern of enrichment compared with that of memory encoding genes (Supplementary Fig. S3a, b). The results suggest that genes related to brain advanced functions are extensively regulated in the twin-peak periods during neocortex development.

### Enrichment of autism and Alzheimer’s disease genes in developmental DEGs

We hypothesized that neurodevelopmental disease genes tend to be differentially regulated in the neocortex during development. As expected, 247 out of 1,007 autism-related genes were differentially expressed in the middle foetal brain (13 pcw to 24 pcw; Benjamini–Hochberg corrected *P* < 0.05). The biased distribution of autism disease genes was not detected in other developmental stages (Fig. 3c). At the early mid-foetal (13 pcw to 19 pcw) developmental stage, 159 autism-related genes were enriched in DEGs among seven regions (Benjamini–Hochberg corrected *P* ≤ 0.014). Of these genes, eleven were differentially expressed in all these regions (Supplementary Table S5).

Additionally, out of 680 Alzheimer’s disease genes, 342 were differentially expressed during development (Benjamini–Hochberg corrected *P* < 0.05). However, unlike the autism disease genes, which tend to be enriched in DEGs of the middle foetal brain, Alzheimer’s disease genes tended to be differentially regulated at late adulthood developmental periods (age ≥ 60 years old). In total, 32 Alzheimer’s disease genes were differentially expressed in the late adulthood period, and 23 of these genes significantly overlapped with DEGs in five regions (OFC, ITC, S1C, VFC, and STC; Benjamini–Hochberg corrected *P* ≤ 0.035; Fig. 3d). Consistent with recent results^20–22^, our analysis highlighted the molecular nature of Alzheimer’s disease at the transcriptomic level.

### Excitatory neurons are more regulated than inhibitory neurons in the prenatal brain

**Fig. 4:**
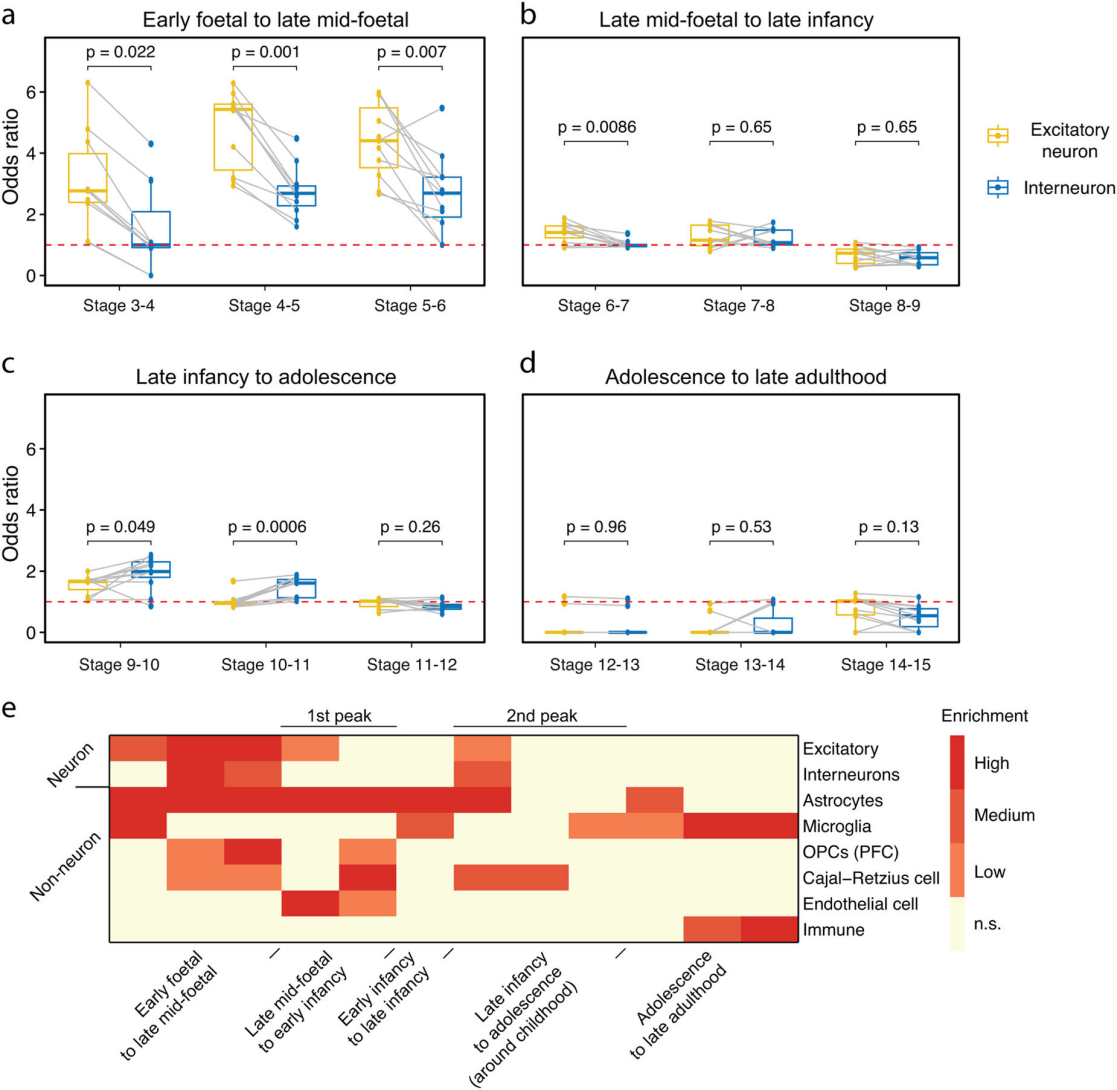
Regulation of different cell types during neocortical development. **a**–**d** Enrichment of excitatory and interneuron markers at four developmental stages: **a** from early foetal to late mid-foetal period, **b** from late mid-foetal to late infancy, **c** from late infancy to adolescence, and **d** from adolescence to late adulthood. Dots in the boxplot represent neocortical subregions. Grey lines link the corresponding neocortical subregions between excitatory neurons and interneurons. **e** Comparison of the enrichment of marker genes from multiple cell types with developmental DEGs. High, *P* < 0.001; Medium, *P* < 0.001, Low, *P* < 0.05. Not enriched, *P* > 0.05

According to a recent study, novel excitatory neurons have evolved while inhibitory neurons are conserved in the evolution of the mammalian neocortex^23^, which indicates the unique role of excitatory neurons in the development of the human cerebral cortex. Therefore, we explored the differential regulation of multiple cell types in different developmental stages^24–26^. In foetal periods, the markers of excitatory neurons were significantly more regulated than were the markers of inhibitory neurons (*P* < 0.05 in all comparisons, Fig. 4a, b). In childhood, slightly more regulations of inhibitory neuron-related markers were found (stage 9–10 and stage 10–11, *P* < 0.05, Fig. 4c). These findings suggest that different computational logic might be developed in the two critical periods. Neither of the two cell types was significantly regulated after adolescence (Fig. 4d). In addition, for non-neuronal cells, astrocytes were strongly regulated at the early stages of development, implying an important role in early brain development (Fig. 4e). Similar patterns were found in markers of Cajal-Retzius cells and oligodendrocyte progenitor cells. In contrast, microglia and immune cells were mainly regulated in late stages (Fig. 4e).

### Modularity relationship links developmental DEGs with functionally important pathways

To understand the modularity features of developmental DEGs at the two peak periods, weighted gene co-expression analysis was performed for each period^27–29^. For networks of the first peak period, we identified 7 out of 19 modules that are enriched with developmental DEGs of the first peak (first peak DEG modules: black, blue, brown, dark orange, light green, tan and turquoise; Benjamini–Hochberg corrected *P* < 8.22 × 10^−5^); among these, six modules were also enriched with developmental DEGs of the second peak (Benjamini–Hochberg corrected *P* < 8.41 × 10^−3^; Supplementary Fig. S4a). Similarly, for the networks of the second peak period, we discovered 15 out of 25 modules enriched with developmental DEGs of the second peak (Benjamini–Hochberg corrected *P* < 2.04 × 10^−4^); among these, six were also enriched with developmental DEGs of the first peak (Benjamini–Hochberg corrected *P* < 5.65 × 10^−5^; Supplementary Fig. S4b, Supplementary Table S6). These highly overlapping modules indicate that similar co-expression regulatory networks are involved in the critical developmental period of the brain.

In addition, to explore whether the DEGs in twin-peak modules are related to the tipping points of brain development, we applied the dynamic network biomarker method^30^. We found dynamic network biomarkers consisting of 1,845 genes and that a critical stage just emerges after childhood, which may imply the transition of the brain to a mature state. By assessing the top linked 300 genes in the networks of the twin peaks, we found that 20.7% (62/300) and 14.3% (43/300) of these genes were dynamic network biomarkers in the first and the second peak network modules, respectively.

Further functional analysis of the top linked genes in those DEG modules revealed that they were relevant to biological pathways such as GABAergic synapse, calcium signalling pathway, axon guidance and synaptic vesicle cycle (Benjamini–Hochberg corrected *P* < 9.32 × 10^−4^; Supplementary Fig. S4c, d). Specifically, the second peak DEG modules were associated with pathways related to learning and memory, such as long-term potentiation (Benjamini–Hochberg corrected *P* = 2.89 × 10^−4^). Moreover, we found a “purple” module in the first peak period and a “green” module in the second peak period, which were both enriched with Parkinson’s disease, Alzheimer’s disease and Huntington’s disease (first peak: Benjamini–Hochberg corrected *P* < 1.27 × 10^−10^; second peak: Benjamini–Hochberg corrected *P* < 3.22 × 10^−4^). Our results imply the intrinsic link of developmental DEGs and those important neurological processes at the network level.

We noticed that both “memory or learning” and “cognition” genes were highly associated with DEG modules in the twin-peak periods, i.e., two modules in the first peak period (blue, Benjamini–Hochberg corrected *P* = 4.31 × 10^−3^; dark orange, Benjamini–Hochberg corrected *P* = 0.0184; Supplementary Fig. S4e, f) and one module in the second peak period (pale turquoise, Benjamini–Hochberg corrected *P* = 2.97 × 10^−4^; Supplementary Fig. S4g and Supplementary Table S7). For example, in the blue module, we found two “cognition” hub genes, *PTN* and *GM2A*; *GM2A* was associated with an ASD-related hub gene *SLC1A2*. Moreover, we found a hub gene, *PLCD3*, which was a dynamic network biomarker and was associated with several ASD-related hub genes, such as *PRODH*, *CAMK2A*, *DOCK10* and *RAPGEF4* (Supplementary Fig. S4e).

Finally, some co-expression modules were enriched with both first peak DEGs and the second peak DEGs. For example, by comparing all the DEG modules between the twin-peak networks, we detected a first-peak DEG module involved in IgG binding (light green, Benjamini–Hochberg corrected *P* = 1.93 × 10^−3^) and a second-peak DEG module related to taste transduction (white, Benjamini–Hochberg corrected *P* = 3.75 × 10^−7^). The functional implications of these modules during brain development need to be further investigated.

### Developmental DEGs in the human early foetal brain are two-fold higher than those in rhesus macaque

Identifying human-specific DEGs is an important topic as it may indicate the molecular basis for the uniqueness of the human brain^3,31,32^. Here, we estimated macaque developmental DEGs by utilizing the recently released non-human primate brain developmental atlas^6^ and compared them with our human developmental DEGs. Due to the limitation of the sampling time points and brain regions in macaques, only samples from the V1C region spanning from the early foetal period to young childhood were included in the final analysis. This comparison of the expression of orthologous genes between human and macaque in this region showed that the number of developmental DEGs in human early foetal brain V1C was two-fold higher than that in macaque V1 (Supplementary Fig. S5a and Supplementary Table S8). Moreover, more genes were differentially regulated in the macaque brain after birth, and the second peak occurred earlier in macaque V1 at adjacent stages of 3 months to 12 months (Supplementary Fig. S5b and Supplementary Tables S8, S9). These results indicate that the transcriptional regulation of early stages is more important to the uniqueness of the human brain.

## Discussion

Our analyses showed that >80% of expressed genes are differentially regulated during the development of the human cerebral neocortex. The distribution of these DEGs is not uniform, with more significant differences in the transcriptome of the human foetal cortex compared with that of the rest of the lifespan, which leads to a waterfall mode regarding the global distribution of developmental DEGs. Furthermore, two bursts of regulation in the transcriptome of neocortex were observed: the first peak, which is the greater one, occurs in the perinatal period, while the second peak of developmental regulation occurs around the childhood period. A large number of genes are involved in these two significant regulatory periods of cortical development, and many of these genes are from similar functional pathways and co-expression networks. The functional analysis of those genes and their related pathways suggested that these two critical periods provide the molecular basis for human neocortical growth and maturation. The twin-peak regulatory patterns were replicated by using an independent dataset containing the lifespan transcriptome of the human prefrontal cortex. Similarly, according to recent research, a large number of genes experience dramatic expressional changes in both late foetal and childhood-adolescence periods^26^. Moreover, among the five typical genes involved in brain-based traits and neuropsychiatric diseases, four of them (*MEF2C*, *SATB2*, *SOX5*, *TCF4*, and *TSHZ3*) were found in our twin-peak gene lists.

Our enrichment analysis of developmental DEGs in neurological disease genes revealed that many disease-related genes, such as autism- or Alzheimer-related genes, are tightly regulated only at some specific developmental stages. However, genes that are potentially important for advanced brain functions, such as resting state-related genes or memory encoding-related genes, tend to be regulated in multiple developmental stages. The differences in the distribution pattern are confirmed by using genes annotated as “learning or memory” and “cognition”, indicating the complexity of long-term regulation of these functional genes during neocortex development. More importantly, the markers of excitatory neurons are more regulated than those of inhibitory neurons in early cortical development. The cell type enrichment pattern of temporal DEGs is consistent with the timeline of neural cell generation and maturation. More interestingly, the markers of astrocytes are enriched in the mid-fetal brain, which may indicate an unexpected role for them in this period. Together, these findings emphasized the importance of excitatory neurons in the uniqueness of the human brain. Finally, the modularity feature of these genes in the twin-peak co-expression networks suggests an intrinsic link among all these different genes involved in the nervous system.

The number of interarea DEGs remains large in different foetal periods of the neocortex and decreases rapidly after birth^11^, which is significantly different from the pattern of developmental DEGs. The developmental synchronization of different cortical regions in this period may be linked to the first regulatory peak observed. Indeed, a large number of overlapping genes exist between developmental and interareal DEGs. Consistent with spatial synchronization^11^, the significant overlap of the developmental DEGs among different neocortex regions suggests that synchronization also occurs temporally during development, which may be of great importance for the correct formation of neural circuits among those regions. Thus, the twin-peak regulation and hourglass mode distribution of DEGs provide the basis for further understanding the global temporal and spatial developmental regulation of the human neocortex.

## Materials and methods

### HBT data

Two microarray datasets on the gene expression of developing human brain and one dataset on developing rhesus monkey brain were used for analysis. The microarray data of human neocortical development were downloaded from the HBT database^10^ (http://hbatlas.org/), which includes 14,047 genes in the downstream computation. The microarray data of the human prefrontal cortex were obtained from previous research^7^, which includes 17,162 genes. The microarray data of the developing brain of rhesus monkey were obtained from^6^, which contains 12,441 genes.

### Temporal and developmental DEG identification

For each neocortical region, temporal DEGs during different development stages were estimated by slightly modifying a previously established method of identification of regional DEGs^11^. First, ANOVA was performed to identify DEGs in the neocortex, PFC, and macaque brain. Then, to control for the false discovery rate (FDR), the significant level (*p* values) from ANOVA test were corrected with the Benjamini and Hochberg procedure for multiple comparisons^33^. The threshold of DEGs was chosen as previously reported^11^ (FDR *Q* value < 0.01, fold change ≥ 2), including the post-mortem interval (PMI) and RNA integrity number (RIN) as technical covariates. For the PFC and macaque datasets, we did not control for covaried factors as neither PMI nor RIN information was provided.

A post hoc Tukey’s HSD test was performed to calculate the differences between each pair of developmental stages in each neocortex subregion. The number of significant comparisons for each stage per neocortex subregion was then used as the number of inter-stage DEG combinations. To investigate the expression changes of the temporal DEG stage by stage, we defined developmental DEGs by the following criteria: in Tukey’s HSD test, the absolute value of the difference between two adjacent developmental stages is ≥1.

### Permutation experiments

Permutation tests were performed to examine the statistical significance of the overlapping developmental DEGs across 11 neocortex regions. For a given adjacent developmental stage, the number of shared DEGs across neocortex regions was calculated. Then, for each simulation, the number of DEGs was resampled to calculate the number of overlapped DEGs. Ten thousand permutation experiments were performed for each stage, and the subsequent *p*-value was determined.

### Functional gene enrichment analysis

An autism-related gene list was obtained from the SFARI database^34^ (10/07/2018, https://gene.sfari.org/database/gene-scoring/). Memory encoding-related genes were collected from previous research^19^. Resting state fMRI-related genes were previously described^17,18^. The learning or memory gene list and cognition gene list were downloaded from AmiGO^35^ (Accession: GO:0007611 and GO:0050890, http://amigo.geneontology.org/amigo/). GO/KEGG enrichment analyses were performed using clusterProfiler package v3.8.1^36^ with R. The enrichment analysis of the functional gene set was performed using Fisher’s exact test with the Benjamini–Hochberg procedure to adjust the p values.

### Weighted gene co-expression network analysis

Weighted gene co-expression network analysis (WGCNA) was performed for the twin-peak regulation periods (periods 6–8 and 10–11), respectively. In total, 226 samples from the first peak and 82 samples from the second peak were included in the computation. Signed co-expression networks were built with the WGCNA package v1.63^28,29^ in R. For all genes included in the analysis, a pairwise correlation matrix was computed; then, an adjacency matrix was calculated by raising the correlation matrix to power 18, according to a scale-free topology criterion^29^. For each pair of genes, a robust measure of network interconnectedness (topological overlap measure) was calculated, and the modules were generated by hybrid dynamic tree cutting^37^. Other parameters used in the analysis were the minimum module size = 50, deepSplit = 4, and the minimum height for merging modules = 0.15.

### Dynamic network biomarker analysis

The dynamic network biomarker genes were identified by modifying a previously established method^30^. Briefly, we first calculated DEGs by *t*-test (FDR corrected) at each development stage against the first development stage. Next, we clustered genes at each stage by Pearson correlations and set the maximum cluster number as 40. For each cluster, four parameters were calculated in each stage: the standard deviation (SD), the average Pearson correlation coefficient (PCC) among the cluster members, the average PCC between the in-cluster genes and out-cluster genes, and the composite index (CI). Finally, we selected the cluster that showed the greatest change in the CI.

### Cell type markers

Cell type marker genes were obtained from recently released data^24–26^. The enrichment analysis of marker genes at each developmental period was performed using Fisher’s exact test, with the Benjamini–Hochberg procedure to adjust the *p* values. To determine the overall differential regulation of each cell type in the 11 neocortical regions, we used the enrichment of marker genes in at least half of all regions as a threshold. For instance, *p* < 0.001 indicates that the marker genes are significantly differentially regulated in >5 of the 11 regions with a significance level of *p* < 0.001.

## Supplementary information

Supplementary Information

supplementary Table S3

supplementary Table S4

supplementary Table S6

supplementary Table S7

supplementary Table S8

supplementary Table S9

## Supplementary information

**Supplementary Information** accompanies the paper at (10.1038/s41421-019-0129-3).
