# Supplementary material for: Twin-peak temporal regulation during human neocortical development
Source: Cell Discov. 2019 Dec 17;5:61. doi: 10.1038/s41421-019-0129-3 (PMC6915741; doi:10.1038/s41421-019-0129-3)
Supplement: Supplementary file 1 — Supplementary Information [file 41421_2019_129_MOESM1_ESM.docx]

**Cell Discovery**

**Supplementary information**

**Twin-peak temporal regulation during human neocortical development**

Wei Wang ^1^, Guang-Zhong Wang^1*^

^1^CAS Key Laboratory of Computational Biology, CAS-MPG Partner Institute for Computational Biology, Shanghai Institute of Nutrition and Health, Shanghai Institutes for Biological Sciences, University of Chinese Academy of Sciences, Chinese Academy of Sciences, Shanghai, 200031, China.

^*^Corresponding to: guangzhong.wang@picb.ac.cn

**Supplementary Figs. S1-S3**

**Supplementary Table S1-S9**

**Supplementary Figures.**

**
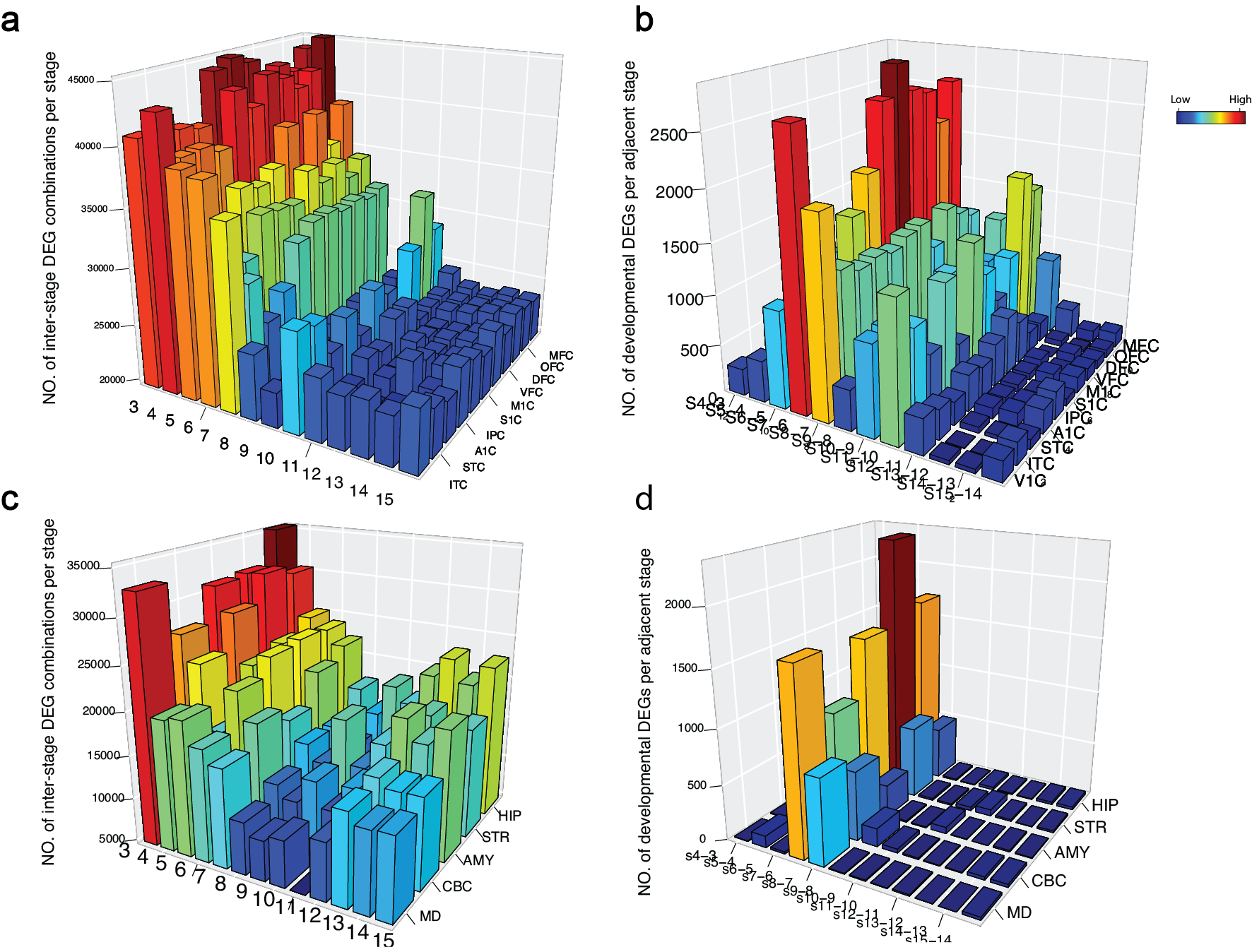
**

**Supplementary Fig. S1 Distribution of temporal and developmental DEGs.**

**a** The permutated number of temporal DEGs in 11 neocortex regions (result of one permutation test).

**b** The permutated number of adjacent developmental DEGs per neocortex subregion (result of one permutation test).

**c** The number of temporal DEGs combinations of 5 non-neocortex regions (MD, mediodorsal nucleus of the thalamus; CBC, cerebellar cortex; AMY, amygdala; STR, striatum; HIP, hippocampus).

**d** The number of adjacent developmental DEGs per non-neocortex region.

**Supplementary Fig. S2 GO enrichment analysis of 1^st^ peak only DEGs, i.e. DEGs found only in the 1^st^ peak but not in the 2^nd^ peak period.**

**
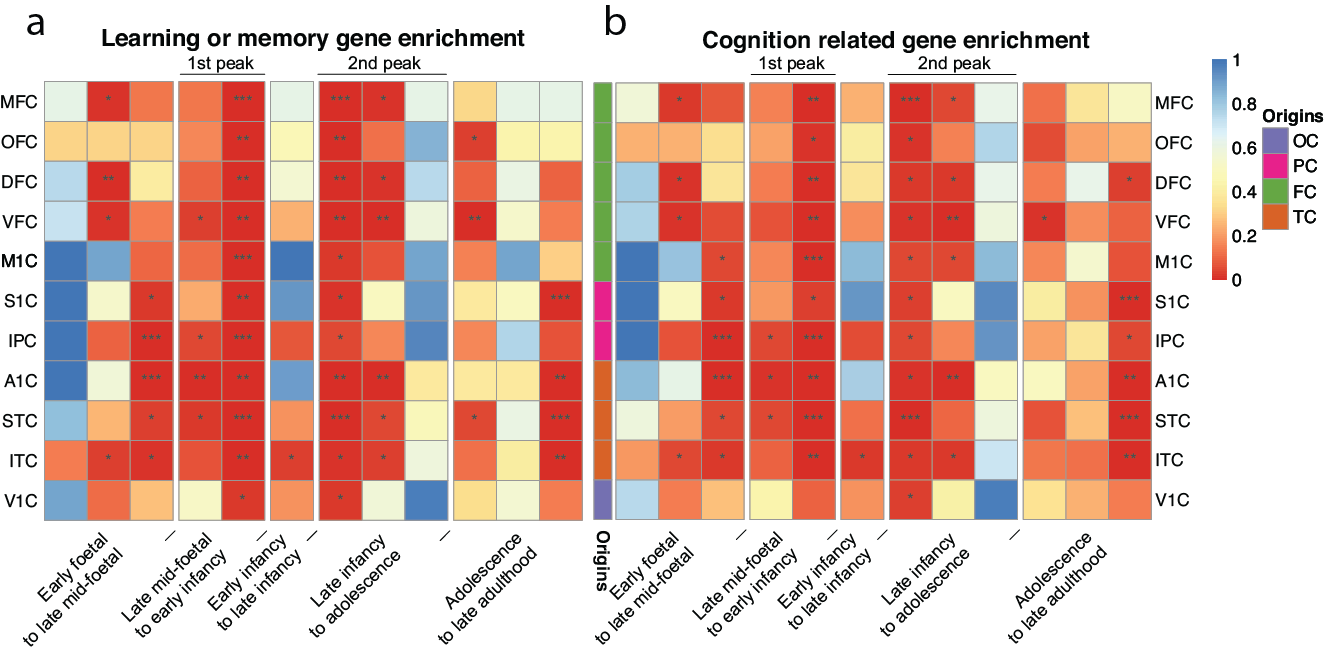
**

**Supplementary Fig. S3 Enrichment of brain functional gene sets with developmental DEGs.**

**a** Learning/memory associated genes are widely enriched in early foetal, late adulthood and twin-peak periods.

**b** Cognition associated genes are widely enriched in early foetal, late adulthood and twin-peak periods.

**
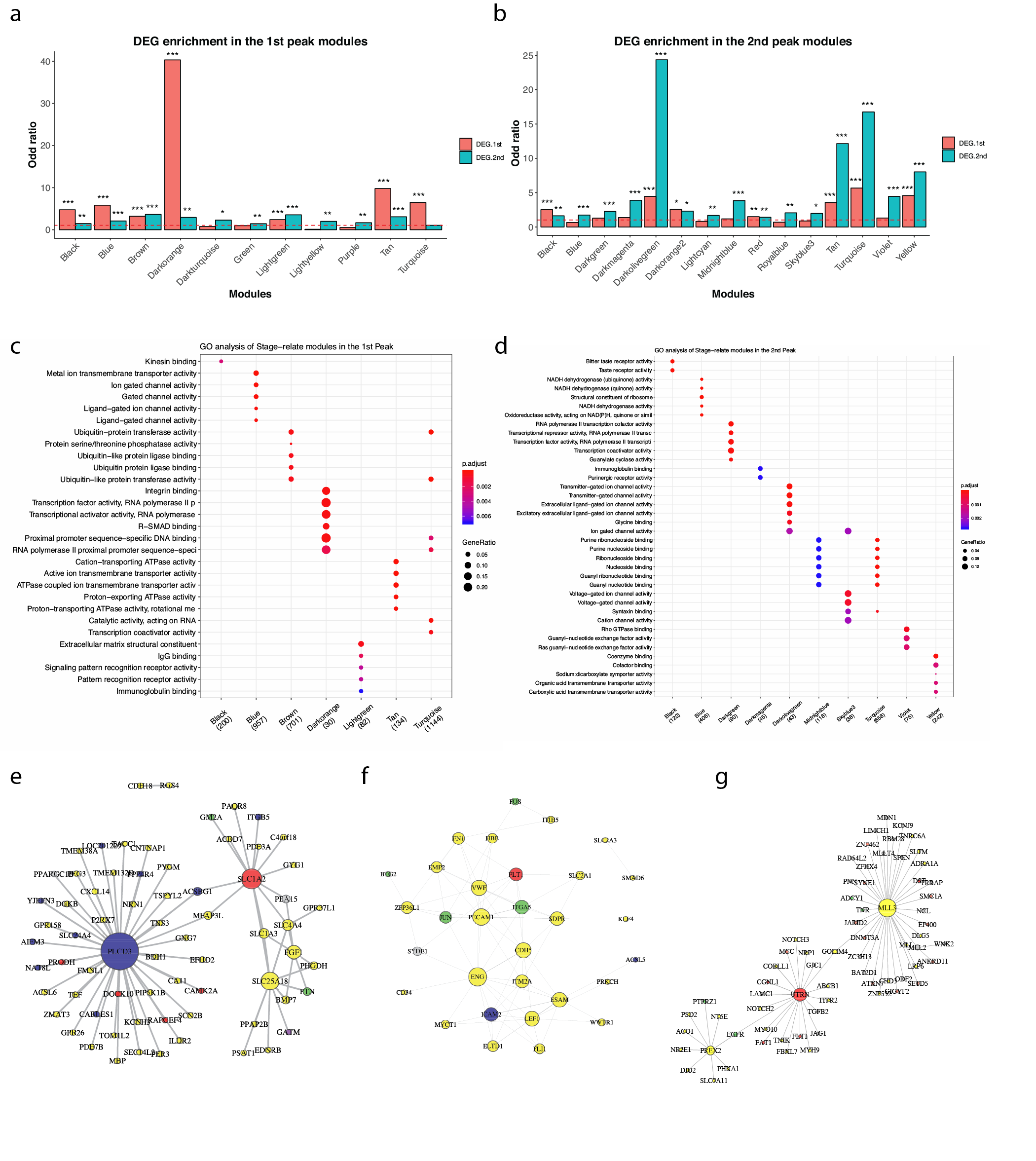
**

**Supplementary Fig. S4 Weighted gene co-expression analysis (WGCNA) of twin-peak periods.**

**a**, **b** Enrichment of developmental DEGs in different co-expression modules in the 1^st^ peak (**a**) and 2^nd^ peak (**b**) periods. The red bar represents DEGs from the 1^st^ peak period, and the green bar represents DEGs from the 2^nd^ peak period. The red dashed line represents the odds ratio of 1. Asterisk on the bar plot means a significant over-enrichment (odds ratio > 1) of the DEGs within a module. * p < 0.05, ** p < 0.01, *** p < 0.001.

**c**, **d** GO enrichment of DEG over enriched modules in the 1^st^ peak (**c**) or 2^nd^ peak (**d**) periods.

**e**-**g** Network graphs of three DEG modules: blue (**e**) and darkorange (**f**) modules from the 1^st^ peak period; magenta module (**g**) from the 2^nd^ peak period. All of the three modules are enriched with the genes annotated under the term “cognition”.

**
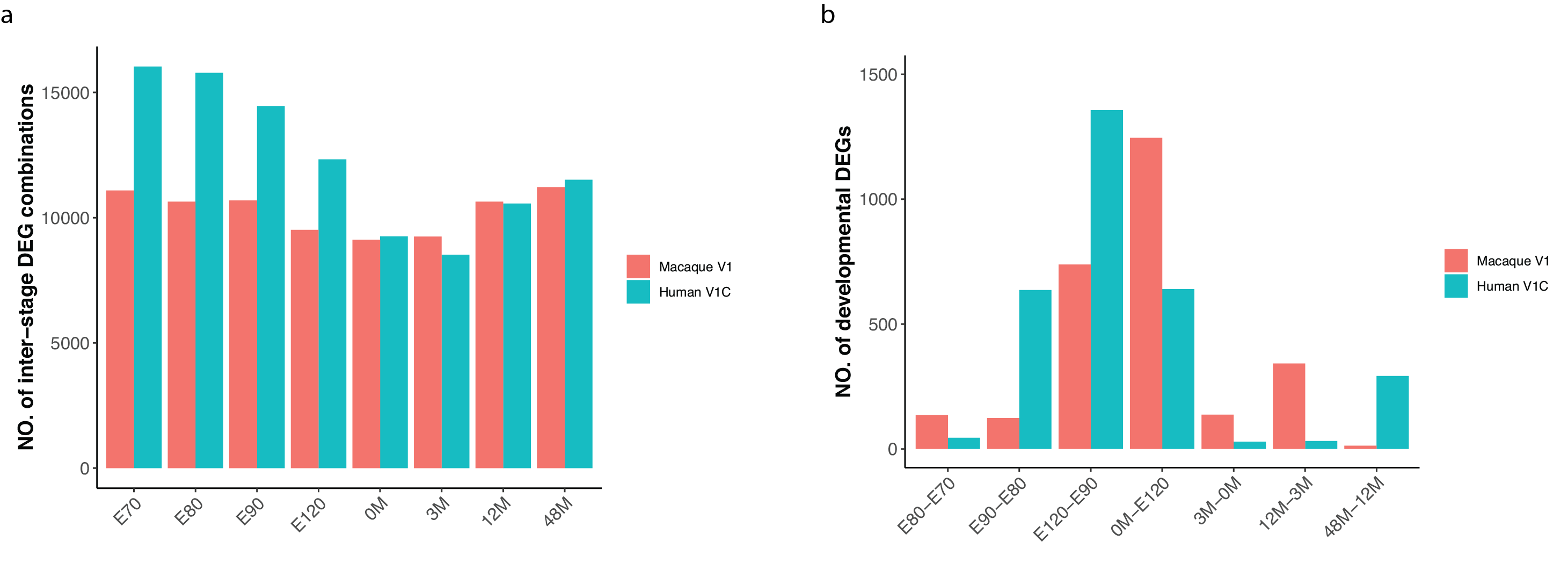
**

**Supplementary Fig. S5 Comparison of human and macaque DEGs.**

**a**, **b** Comparison of the number of temporal DEG combinations (**a**) and developmental DEGs

between two adjacent stages (**b**) of human V1C and macaque V1.

**Supplementary tables**

**Supplementary Table S1. Number of temporal DEGs of each neocortex subregion.**

This table summarizes the number of temporal DEGs, and the proportion of DEGs in all expressed genes for each neocortex subregion. A total of 11,771 genes were differentially expressed in at least one neocortex subregion.

**Supplementary Table S2. Number of developmental DEGs at each adjacent developmental stage.**

This table shows the number of developmental DEGs that were identified at adjacent developmental stages for each neocortex subregion.

**Supplementary Table S3. List of developmental DEGs in the human prefrontal cortex.**

This table lists the developmental DEGs at adjacent developmental stages in the human prefrontal cortex. “Yes” represents DEG, and “No” represents non-DEG at each adjacent-stage.

**Supplementary Table S4. List of twin-peak DEGs.**

List of all DEGs that were differentially expressed in the twin-peak periods. 1^st^ peak period includes adjacent-stages 6-7 and 7-8; 2^nd^ peak period includes adjacent-stages 9-10, 10-11 and 11-12.

**Supplementary Table S5. Developmental and regional overlapped DEGs.**

This table summarizes the number of overlapped genes between developmental DEGs and regional DEGs.

**Supplementary Table S6. Enrichment of DEGs in WGCNA modules detected in twin-peak periods.**

This table lists the enrichment of DEGs from 1^st^ / 2^nd^ peak periods in the WGCNA modules of the twin-peak periods, respectively.

**Supplementary Table S7. Enrichment of brain functional gene set in the twin-peak periods related WGCNA modules.**

This table shows the statistics of Fisher’s exact test for the enrichment analysis of functional gene sets in the WGCNA modules from twin-peak periods.

**Supplementary Table S8. Comparison of human and macaque developmental DEGs.**

This table summarizes the number of developmental DEG combination and the number of developmental DEGs between human and macaque one-to-one orthologous.

**Supplementary Table S9. List of human and macaque one-to-one orthologous developmental DEGs.**

This table lists the developmental DEGs of one-to-one orthologous in human V1C and macaque V1. “Yes” represents DEG, and “No” represents non-DEG at each adjacent-stage.

**Supplementary Table S1. Number of temporal DEGs of each neocortex subregion**

|  | Counts (Temporal DEGs) | Proportion |
| --- | --- | --- |
| V1C | 9654 | 69.78% |
| ITC | 8696 | 62.86% |
| STC | 8735 | 63.14% |
| A1C | 8639 | 62.45% |
| IPC | 9091 | 65.71% |
| S1C | 9348 | 67.57% |
| M1C | 9217 | 66.63% |
| VFC | 9135 | 66.03% |
| DFC | 8996 | 65.03% |
| OFC | 9393 | 67.90% |
| MFC | 9415 | 68.06% |
| Sum | 11771 | 85.09% |

**Supplementary Table S2. Number of developmental DEGs at each adjacent developmental stage**

|  | S4-S3 | S5-S4 | S6-S5 | S7-S6 | S8-S7 | S9-S8 | S10-S9 | S11-S10 | S12-S11 | S13-S12 | S14-S13 | S15-S14 |
| --- | --- | --- | --- | --- | --- | --- | --- | --- | --- | --- | --- | --- |
| V1C | 174 | 585 | 429 | 3142 | 2061 | 294 | 646 | 1159 | 581 | 46 | 30 | 270 |
| ITC | 149 | 392 | 272 | 1828 | 1337 | 155 | 676 | 784 | 292 | 29 | 26 | 85 |
| STC | 99 | 511 | 332 | 1465 | 1281 | 150 | 704 | 851 | 345 | 35 | 45 | 95 |
| A1C | 90 | 485 | 142 | 1525 | 1346 | 182 | 427 | 1040 | 338 | 59 | 61 | 81 |
| IPC | 40 | 679 | 197 | 2275 | 1314 | 177 | 749 | 651 | 355 | 35 | 56 | 97 |
| S1C | 40 | 1002 | 930 | 2812 | 1363 | 256 | 801 | 1296 | 600 | 46 | 23 | 74 |
| M1C | 43 | 1031 | 1015 | 2743 | 1415 | 177 | 586 | 851 | 539 | 26 | 63 | 82 |
| VFC | 71 | 649 | 180 | 2439 | 1220 | 170 | 712 | 983 | 395 | 35 | 33 | 139 |
| DFC | 76 | 613 | 345 | 2282 | 1245 | 132 | 944 | 1053 | 199 | 22 | 39 | 45 |
| OFC | 181 | 799 | 264 | 2278 | 1151 | 245 | 1343 | 1882 | 193 | 25 | 26 | 69 |
| MFC | 288 | 646 | 552 | 2477 | 1289 | 142 | 1038 | 1663 | 527 | 45 | 52 | 118 |

**Supplementary Table S5. Developmental and regional overlapped DEGs**

|  | V1C | ITC | STC | A1C | IPC | S1C | M1C | VFC | DFC | OFC | MFC |
| --- | --- | --- | --- | --- | --- | --- | --- | --- | --- | --- | --- |
| Stage3 | 905 | 794 | 720 | 676 | 637 | 679 | 686 | 741 | 762 | 827 | 825 |
| Stage4 | 932 | 722 | 658 | 650 | 742 | 705 | 733 | 764 | 745 | 849 | 925 |
| Stage5 | 1049 | 838 | 711 | 936 | 771 | 902 | 977 | 771 | 888 | 874 | 1053 |
| Stage6 | 730 | 575 | 579 | 541 | 553 | 592 | 595 | 567 | 611 | 628 | 604 |
| Stage7 | 273 | 208 | 175 | 159 | 150 | 173 | 194 | 185 | 192 | 209 | 191 |
| Stage8 | 18 | 16 | 12 | 14 | 11 | 14 | 13 | 14 | 16 | 16 | 15 |
| Stage9 | 14 | 11 | 11 | 9 | 9 | 13 | 11 | 9 | 11 | 10 | 15 |
| Stage10 | 4 | 4 | 6 | 3 | 3 | 5 | 6 | 4 | 5 | 6 | 6 |
| Stage11 | 4 | 2 | 1 | 2 | 1 | 2 | 1 | 1 | 2 | 2 | 2 |
| Stage12 | 115 | 71 | 50 | 52 | 62 | 56 | 50 | 62 | 75 | 69 | 88 |
| Stage13 | 256 | 196 | 85 | 116 | 88 | 105 | 128 | 97 | 97 | 112 | 205 |
| Stage14 | 265 | 181 | 138 | 124 | 152 | 148 | 135 | 161 | 175 | 168 | 201 |
| Stage15 | 535 | 258 | 179 | 265 | 224 | 258 | 283 | 237 | 276 | 289 | 480 |
